# Integrative Bioinformatics-Guided Analysis of Glomerular Transcriptome Implicates Potential Therapeutic Targets and Pathogenesis Mechanisms in IgA Nephropathy

**DOI:** 10.3390/bioengineering12101040

**Published:** 2025-09-27

**Authors:** Tiange Yang, Mengde Dai, Fen Zhang, Weijie Wen

**Affiliations:** 1 Department of Food Science and Engineering, College of Life Science and Technology, Jinan University, Guangzhou 510632, China; tgyang@stu2024.jnu.edu.cn (T.Y.); demon@stu.jnu.edu.cn (M.D.); 2 Guangdong Institute of Gastroenterology, The Sixth Affiliated Hospital of Sun Yat-sen University, Sun Yat-sen University, Guangzhou 510655, China; 3 Key Laboratory of Human Microbiome and Chronic Diseases, Sun Yat-sen University, Ministry of Education, Guangzhou 510655, China; 4 Guangdong Provincial Key Laboratory of Colorectal and Pelvic Floor Diseases, The Sixth Affiliated Hospital of Sun Yat-sen University, Sun Yat-sen University, Guangzhou 510655, China; 5 Biomedical Innovation Center, The Sixth Affiliated Hospital, Sun Yat-sen University, Guangzhou 510655, China

**Keywords:** IgA nephropathy, disease biomarker, bioinformatics, machine learning

## Abstract

(1) Background: IgA nephropathy (IgAN) is a leading cause of chronic kidney disease worldwide. Despite its prevalence, the molecular mechanisms of IgAN remain poorly understood, partly due to limited research scale. Identifying key genes involved in IgAN’s pathogenesis is critical for novel diagnostic and therapeutic strategies. (2) Methods: We identified differentially expressed genes (DEGs) by analyzing public datasets from the Gene Expression Omnibus. Gene Ontology and Kyoto Encyclopedia of Genes and Genomes analyses were performed to elucidate the biological roles of DEGs. Hub genes were screened using weighted gene co-expression network analysis combined with machine learning algorithms. Immune infiltration analysis was conducted to explore associations between hub genes and immune cell profiles. The hub genes were validated using receiver operating characteristic curves and area under the curve. (3) Results: We identified 165 DEGs associated with IgAN and revealed pathways such as IL-17 signaling and complement and coagulation cascades, and biological processes including response to xenobiotic stimuli. Four hub genes were screened: three downregulated (FOSB, SLC19A2, PER1) and one upregulated (SOX17). The AUC values for identifying IgAN in the training and testing set ranged from 0.956 to 0.995. Immune infiltration analysis indicated that hub gene expression correlated with immune cell abundance, suggesting their involvement in IgAN’s immune pathogenesis. (4) Conclusion: This study identifies FOSB, SLC19A2, PER1, and SOX17 as novel hub genes with high diagnostic accuracy for IgAN. These genes, linked to immune-related pathways such as IL-17 signaling and complement activation, offer promising targets for diagnostic development and therapeutic intervention, enhancing our understanding of IgAN’s molecular and immune mechanisms.

## 1. Introduction

IgA nephropathy (IgAN) is a predominant form of glomerulonephritis in humans [1], serving as a significant contributor to chronic kidney disease. Approximately 40% of patients progress to end-stage renal disease within two decades of diagnosis [2]. Despite recent advances in identifying clinical biomarkers and proposing hypotheses for IgAN’s pathogenesis, the underlying molecular mechanisms remain poorly understood [3,4]. This knowledge gap highlights the need for comprehensive studies to uncover key genes and pathways involved in IgAN, paving the way for novel diagnostic and therapeutic strategies.

Many studies have been devoted to the discovery of diagnostic markers for IgAN, such as those by Serino et al., who identified six significantly upregulated miRNAs whose regulated genes, GALNT2 and C1GALT1, have been identified as potential biomarkers to predict the incidence of IgAN [5,6]. miRNAs have also been used to monitor disease progression in patients with IgAN [7]. However, limitations in research scale (e.g., small sample sizes) have hindered the identification of critical genes driving IgAN development and progression, necessitating further investigation.

Bioinformatics approaches, integrating large-scale transcriptional data with advanced analytical tools, have emerged as powerful methods to elucidate disease mechanisms. These approaches have successfully identified immune-related genes in diabetic nephropathy, hub genes in membranous nephropathy, and key molecular targets in hypertensive nephropathy [8,9,10,11]. Despite these advances, the molecular landscape of IgAN, including its key regulatory genes and signaling pathways, remains underexplored. Furthermore, machine-learning integration of multi-omics data is accelerating genome-based precision medicine for rare genetic disorders [12,13]. Building on this paradigm, we hypothesized that an integrated machine learning–bioinformatics workflow could uncover robust biomarkers and therapeutic targets for IgA nephropathy, a disease with limited genomic insight.

In this study, we analyzed five independent microarray datasets from the Gene Expression Omnibus (GEO) to identify differentially expressed genes (DEGs) between IgAN patients and healthy controls. We performed Gene Ontology (GO) and Kyoto Encyclopedia of Genes and Genomes (KEGG) enrichment analyses to uncover key biological processes and signaling pathways. Hub genes were identified via weighted gene co-expression network analysis (WGCNA) combined with least absolute shrinkage and selection operator (LASSO) regression and random forest (RF) machine learning. We validated their diagnostic performance using receiver operating characteristic (ROC) curves and explored immune-related mechanisms through immune infiltration analysis. Our work aims to reveal novel molecular targets and immune signatures to advance IgAN diagnosis and therapy.

## 2. Materials and Methods

### 2.1. Preparation of Data and Identification of Differentially Expressed Genes

Microarray datasets relating to IgAN were retrieved from the National Center for Biotechnology Information’s Gene Expression Omnibus (GEO) database (https://www.ncbi.nlm.nih.gov/geo/ (accessed on 22 February 2024)) by using the search term “IgA nephropathy” The datasets were selected based on the following criteria: (1) expression profiling using microarrays, (2) glomerular tissue as the attribute, and (3) Homo sapiens as the organism. Out of the available datasets, five gene expression profiles—GSE99339, GSE37460, GSE32591, GSE93798, and GSE104948—were deemed eligible for analysis [14,15,16,17,18]. Together, these datasets encompass a cohort of 100 IgAN patients and 84 healthy controls. Datasets GSE200828, GSE108113 and GSE99340 were utilized as the validating set. Initially, the probe matrix was extracted using the “GEOquery” package in R, and the probes were subsequently annotated with gene symbols using the corresponding annotation files. In cases where multiple probes were associated with the same gene, the gene’s expression value was determined by calculating the average across all probes. Given that the datasets originated from distinct platforms or laboratories, the “limma” package in R was employed to adjust for these variations across platforms. Subsequently, the “limma” package was utilized to identify differentially expressed genes (DEGs) between IgAN patients and healthy controls. To select DEGs, a stringent criterion was applied: a *p*-value < 0.05 and an absolute log fold change (log_2_FC) > 1.

### 2.2. Enrichment Analysis

The biological processes of the DEGs were further analyzed using the “clusterProfiler” package in R. This package facilitated the enrichment analysis of Gene Ontology (GO) terms and Kyoto Encyclopedia of Genes and Genomes (KEGG) pathways [19]. The GO analysis provided functional annotations by grouping genes into categories based on their associated biological processes, cellular components, and molecular functions. In contrast, the KEGG analysis identified significantly enriched signaling pathways. For both GO and KEGG enrichment analysis, a *p*-value threshold of less than 0.05 was considered statistically significant, indicating the observed enrichment of certain biological processes or pathways among the DEGs were not due to random chance.

### 2.3. Construction of the Weighted Gene Co-Expression Network Analysis

To explore co-expression patterns among genes and identify clusters with similar expression profiles in IgAN, we employed the weighted gene co-expression network analysis (WGCNA) approach using the “WGCNA” package in R. This method constructs a network in which genes are connected based on their pairwise correlation, with connection strength weighted by the correlation coefficient. To determine an appropriate soft threshold power (β), which controls the scale of the network, we utilized the “pickSoftThreshold” function. This function helps identify a β value that satisfies the scale-free topology criterion, indicating that the network is organized with a small number of highly connected genes. Once the optimal β value was determined, we constructed the gene co-expression network using the one-step method. Modules, or clusters, of highly correlated genes were then identified within this network. Each module consisted of at least 100 genes, ensuring that the modules were statistically robust. To identify modules closely associated with IgAN, we calculated correlation coefficients between the module eigengenes (representative of the expression profile of the entire module) and the IgAN phenotype. Additionally, to assess the connection between gene modules and IgAN phenotype, we evaluated gene significance (Gs) and module membership (Mm) values. Gs measures the association between individual genes and the IgAN phenotype, while Mm quantifies the degree of similarity between a gene’s expression profile and the module eigengene. By combining Gs and Mm, we identified genes within modules that are both highly connected and significantly associated with IgAN, providing insights into the key genes and pathways involved in this disease.

### 2.4. Screening of Hub Genes by LASSO Regression and Random Forest Model

To identify hub genes associated with IgAN, we employed a multi-step approach that combined differential expression analysis, WGCNA and machine learning techniques.

First, we utilized the “caret” package in R to divide the data into training and testing groups. This division was conducted in a ratio of 7:3, ensuring that both groups were representative of the overall distribution. The “createDataPartition” function within the “caret” package facilitated random sampling, ensuring even distribution across the two groups. Next, we identified candidate hub genes by intersecting the DEGs and WGCNA results. This approach helped us narrow down the list of genes to those that were both differentially expressed and highly correlated within the gene co-expression network. To prioritize these candidate hub genes further, we employed the Least Absolute Shrinkage and Selection Operator (LASSO) regression. This machine learning algorithm, implemented using the “glmnet” package in R, allows us to simultaneously perform variable selection and complexity regularization. The λ parameter in LASSO regression controls the complexity level of the model, with higher values of λ resulting in a reduced number of selected genes. Through ten-fold cross-validation, we determined the optimal value of λ. Additionally, we constructed a random forest (RF) model using the “randomforest” package in R. This machine learning approach employs multiple independent decision trees to predict classification or regression outcomes. We calculated the MeanDecreaseGini scores for each candidate hub gene and selected those with scores greater than 3. This step helped us identify the most influential genes in the random forest model. By combining these approaches, we identified a set of key genes that are both differentially expressed and highly correlated within the gene co-expression network, and are also important for diagnosing IgAN using machine learning models. These genes provide valuable insights into the biological mechanisms underlying IgAN and could serve as potential targets for further investigation and therapeutic development.

### 2.5. Validation of Hub Genes in IgAN Using ROCs

To evaluate the accuracy of the hub genes identified through machine learning, we generated receiver operating characteristic (ROC) curves, comparing IgAN patients with healthy controls. The area under the ROC curve (AUC) is a metric used to assess the predictive power of a model, with higher AUC values indicating greater accuracy. By calculating the AUC for each hub gene, we assessed their performance in discriminating between IgAN patients and healthy controls. To further validate the effectiveness of the hub genes, we also applied the AUC to the testing set. This allowed us to evaluate the generalizability of our findings. By comparing AUC values across different sets, we gained insights into the consistency and reliability of our hub genes in diagnosing IgAN.

### 2.6. In Silico Estimation of Immune Infiltration Patterns from Transcriptomic Profiles

The CIBERSORTx algorithm is a widely used and reliable tool for analyzing the expression matrix of 22 human immune cell subtypes [20]. In our study, we computationally estimated the relative proportions of immune cell types from publicly available datasets from GEO using the algorithm. This approach is based on the assumption that the expression profiles of immune cells can serve as signatures to deconvolute mixed cell populations. To ensure the accuracy of the results, we employed a rigorous approach by performing 1000 calculations. By applying the CIBERSORTx algorithm to IgAN samples and healthy controls, our study aimed to gain insights into the immune cell composition of these groups and identify any differences that may be associated with IgAN.

### 2.7. Correlation Analysis Between Hub Genes and Infiltrating Immune Cells

Correlation analysis between hub genes and immune cells was performed using the “Hmisc” package in R.

## 3. Results

### 3.1. Identification of DEGs Between IgAN and Healthy Controls

**Figure 1:**
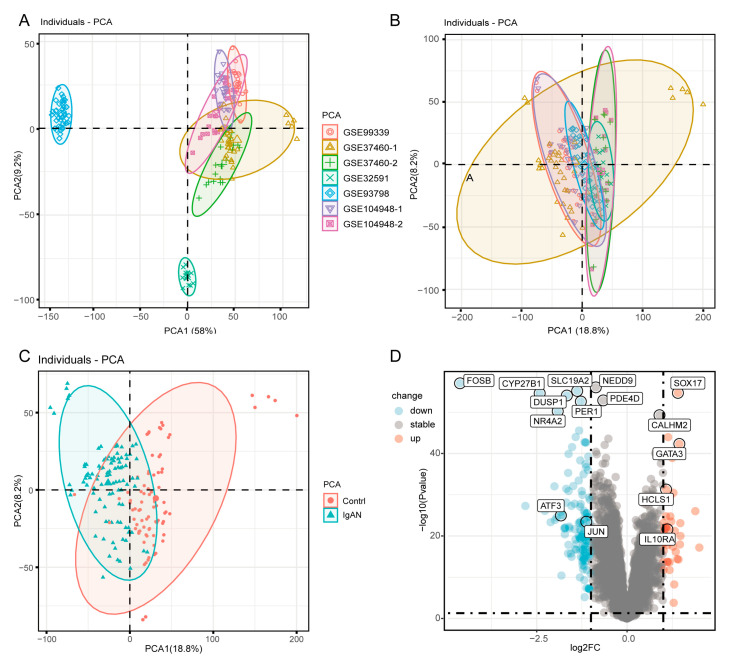
Identification of DEGs between IgAN and healthy controls. (**A**) The principal component analysis (PCA) illustrates the batch effects among the GEO datasets. (**B**) PCA results after batch effect correction. (**C**) PCA demonstrates the segregation between IgAN and healthy controls. (**D**) Volcano plot shows differentially expressed genes (DEGs) between IgAN and healthy controls, with upregulated and downregulated genes highlighted.

Microarray datasets GSE99339, GSE37460, GSE32591, GSE93798 and GSE104948 were retrieved from the GEO database, encompassing a cohort of 100 patients with IgA nephropathy (IgAN) and 84 healthy controls. The expression profiles showed consistency across the samples, achieved through batch effect minimization using the R package (R version 4.3.2), which is essential for advancing subsequent analyses (Figure 1A,B). A principal component analysis (PCA) conducted on the pooled samples revealed distinct segregation between IgAN patients and the healthy controls (Figure 1C). This analysis provides a valuable overview of the underlying biological variations within the dataset. Further exploration of differentially expressed genes (DEGs) identified 165 DEGs, with 122 downregulated such as FOSB, PER1, SLC19A2, DUSP1, ATF3 and JUN being consistent with previous studies [21,22,23] and 43 upregulated genes such as SOX17, GATA3, IL10RA, HCLS1 being related to IgAN [24,25] (Figure 1D). These findings provide valuable insights into the molecular mechanisms underlying IgAN pathogenesis and may serve as potential targets for therapeutic and diagnostic interventions.

### 3.2. Function Enrichment Analysis

**Figure 2:**
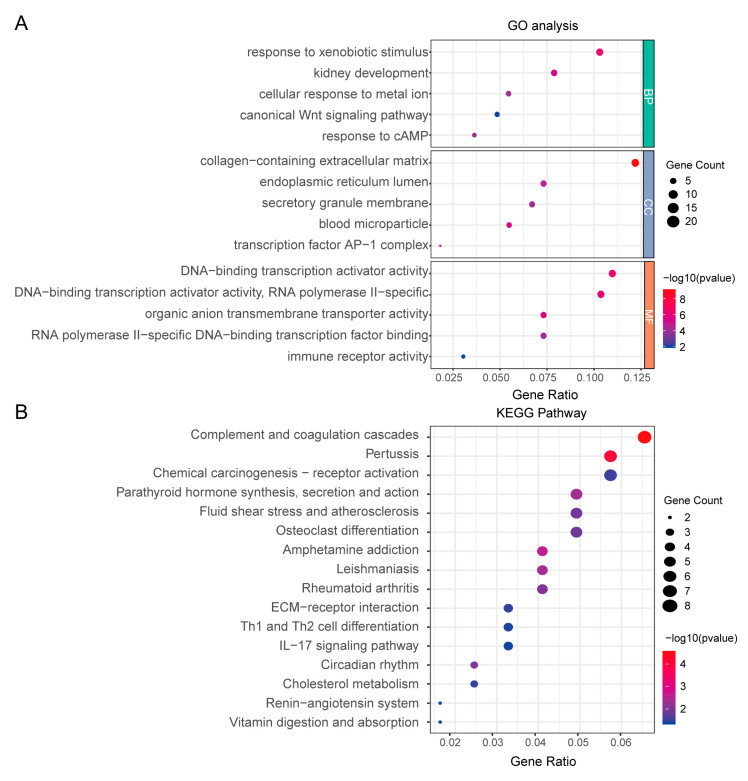
Enrichment analysis of DEGs. (**A**) GO enrichment analysis. (BP: biological process; CC: cell composition; MF: molecular function.) (**B**) KEGG enrichment analysis.

To investigate the biological functions of the identified DEGs, we performed Gene Ontology (GO) enrichment analysis. The important five enriched biological process (BP) terms included response to xenobiotic stimulus, response to cAMP, cellular response to metal ion, kidney development and canonical Wnt signaling pathway. Significant cellular component (CC) terms were associated with the collagen-containing extracellular matrix, blood microparticle, transcription factor AP-1 complex, endoplasmic reticulum lumen and secretory granule membrane. In the molecular function (MF) category, notable enrichments included DNA-binding transcription activator activity, organic anion transmembrane transporter activity, RNA polymerase II-specific DNA-binding transcription factor binding and immune receptor activity (Figure 2A). Kyoto Encyclopedia of Genes and Genomes (KEGG) pathway analysis further identified significantly enriched pathways in IgAN patients compared with healthy controls. These pathways encompassed diverse biological processes, such as the IL-17 signaling pathway, Th1 and Th2 cell differentiation, and complement and coagulation cascades, among others (Figure 2B). Collectively, these findings highlight the pivotal role of immune regulation in the pathogenesis of IgAN.

### 3.3. Construction of WGCNA Network of IgAN

**Figure 3:**
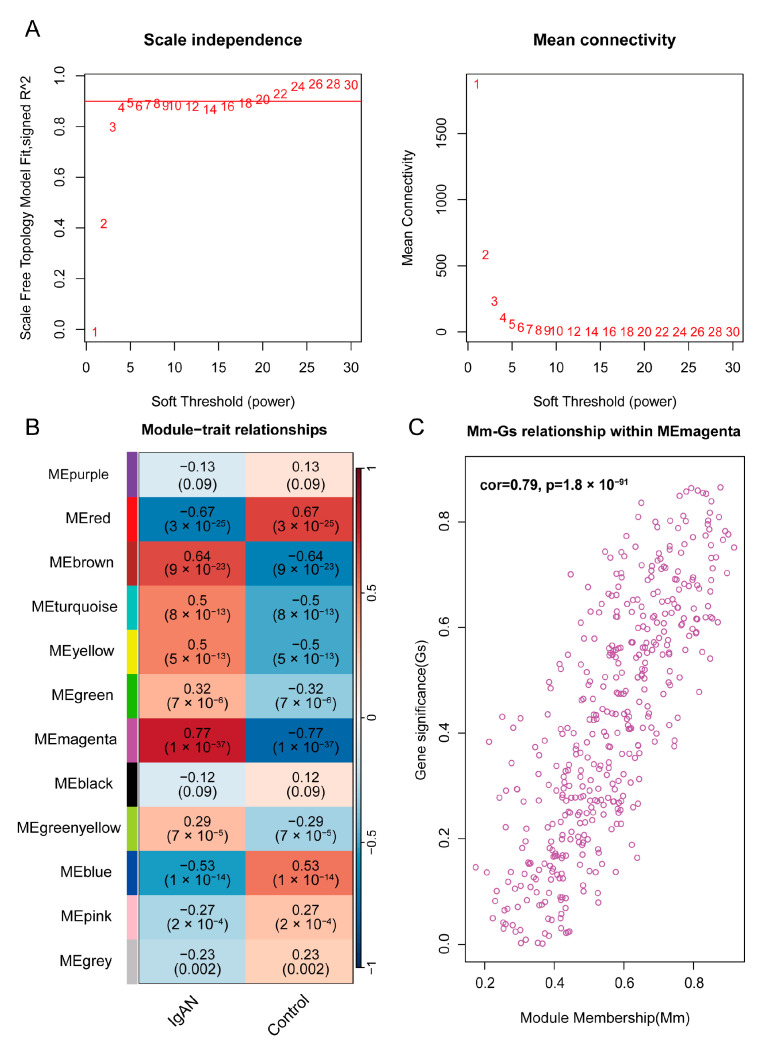
Construction of WGCNA network of IgAN. (**A**) Soft threshold selection for WGCNA to ensure a scale-free topology. The red line represents the selected scale-free fitting index values, while the red numbers represent different soft thresholds. (**B**) Heatmap of the relationships between gene modules and IgAN phenotype. (**C**) Relationship between module membership (Mm) and gene significance (Gs) within the “MEmagenta” module.

To gain a more comprehensive understanding of the genes associated with the IgAN phenotype, we conducted weighted gene co-expression network analysis (WGCNA) on IgAN and healthy controls. The soft threshold was optimized to 4 to ensure a scale-free topology for the network, as shown by the scale-free topology model fit and mean connectivity results (Figure 3A). This approach enabled the construction of a gene hierarchy clustering dendrogram, revealing distinct gene modules with similar co-expression patterns. To pinpoint modules closely related to IgAN, we performed correlation analysis between each module and the IgAN phenotype. The analysis identified the “MEmagenta” module, consisting of 423 genes, as most clinically relevant to IgAN. This finding was supported by a strong correlation coefficient of 0.77 and a highly significant *p*-value of 1 × 10^−37^, indicating a robust association between module feature values and IgAN phenotype (Figure 3B). Furthermore, a significant correlation was observed between gene significance (Gs) and module membership (Mm) within the “MEmagenta” module, with a correlation coefficient of 0.79 and a *p*-value of 1.8 × 10^−91^ (Figure 3C). These results suggest that the “MEmagenta” module may play a key role in IgAN pathogenesis.

### 3.4. Identification and Validation of Hub Genes in IgAN

**Figure 4:**
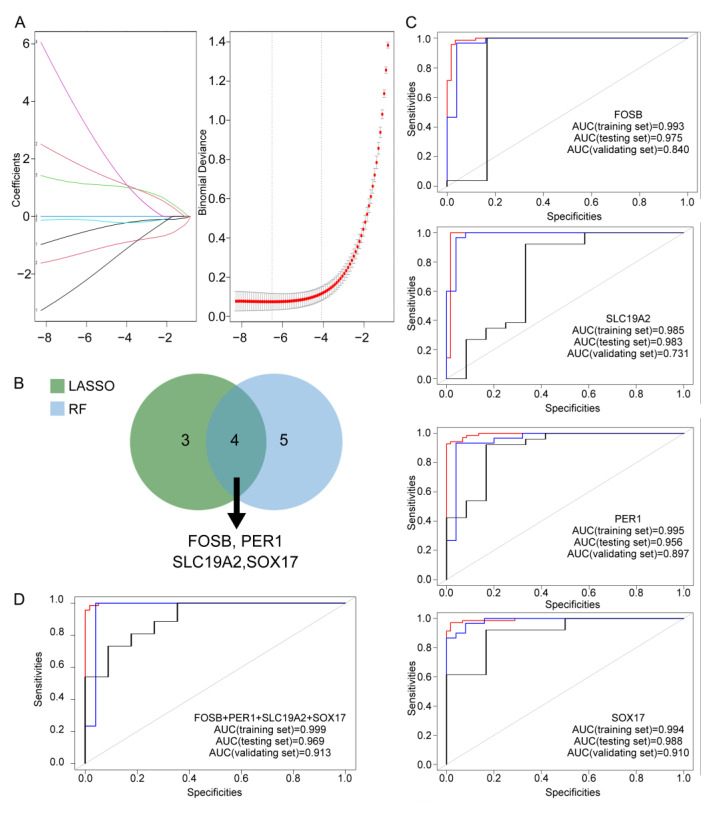
Identification and validation of hub genes in IgAN. (**A**) Selection of the lambda parameter in LASSO regression. Left panel shows the variation trend of coefficients of different features with increasing regularization parameter λ. The numbers in front of the line identify feature number. Right panel shows the variation of model deviation with respect to λ, with red dots representing deviation values and error bars representing standard errors. (**B**) Venn diagram of hub gene selection based on overlap between LASSO and random forest models. (**C**) ROC curves for each hub gene illustrate their diagnostic performance in distinguishing IgAN in training set, testing set and validating set. (**D**) ROC curves for the combined four hub genes illustrate their performance in distinguishing IgAN in training set, testing set and validating set. The red line represents the ROC curve of the training set, the blue line represents that of the testing set, and the black line represents that of the validating set.

To identify the most relevant genes associated with IgAN as hub genes within the WGCNA derived “MEmagenta” module, we employed two robust machine learning techniques: LASSO regression and random forest analysis. LASSO regression identified a subset of seven genes that best predicted the IgAN phenotype (Figure 4A). In parallel, random forest analysis, using a MeanDecreaseGini threshold of ≥3, identified nine highly influential genes. Comparison of the two methods revealed four genes—FOSB, SLC19A2, SOX17, and PER1—that were consistently selected as key predictors by both approaches (Figure 4B). These four genes were designated as the pivotal hub genes associated with IgAN. We next evaluated the diagnostic performance of these hub genes by calculating the area under the receiver operating characteristic curve (AUC) in both training and testing datasets. Each gene demonstrated excellent discriminatory power, with AUC values ranging from 0.956 to 0.995 (Figure 4C). When the four hub genes were combined into a predictive model, the AUC reached 0.999 in the training set and 0.969 in the testing set (Figure 4D). Further validation of the model was conducted using additional datasets of which displayed relatively high AUCs (Figure 4C,D). Generally, these findings highlight the strength of integrating network analysis with machine learning to identify robust biomarkers in complex diseases such as IgAN. The four hub genes identified here represent promising candidates for further mechanistic studies and potential therapeutic targeting.

### 3.5. In Silico Analysis of Immune Infiltration in IgAN

**Figure 5:**
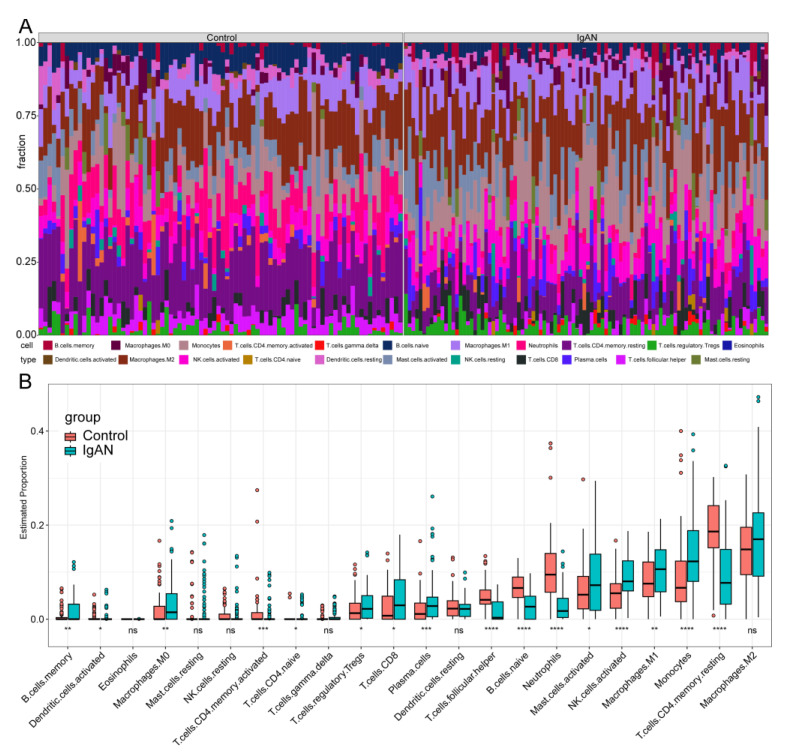
Immune infiltration in IgAN and healthy control samples. (**A**) Proportional distribution of immune cells in each sample. (**B**) Comparison of immune cell abundance between healthy controls and IgAN. (*: *p* < 0.05, **: *p* < 0.01, ***: *p* < 0.001, ****: *p* < 0.0001, ns: not significant).

The in silico immune infiltration analysis conducted on IgAN patients revealed significant findings. When comparing immune cell infiltration in the glomerulus of IgAN patients with that of healthy controls, statistically significant differences were observed. In the IgAN, there was a marked increase in the infiltration of specific immune cell types. Notably, there was a marked elevation in memory B cells, M0 macrophages, naive CD4+ T cells, regulatory T cells (Tregs), CD8+ T cells, plasma cells, activated mast cells, activated NK cells, M1 macrophages, and monocytes (Figure 5A,B). These results suggest that these immune cells play a critical role in the pathogenesis of IgAN. Conversely, certain immune cell types were found to be significantly reduced in the IgAN included activated dendritic cells, activated CD4+ memory T cells, follicular helper T cells, naive B cells, neutrophils, and resting CD4+ memory T cells (Figure 5A,B).

Altogether, these findings indicate that the altered immune cell profile in IgAN glomeruli is potentially characterized by expansion of pro-inflammatory and effector cell subsets alongside the depletion of specific regulatory or memory populations. Further studies are warranted to elucidate their precise functions and interactions in IgAN pathogenesis.

### 3.6. Correlation of Hub Genes with Infiltrating Immune Cells in IgAN

**Figure 6:**
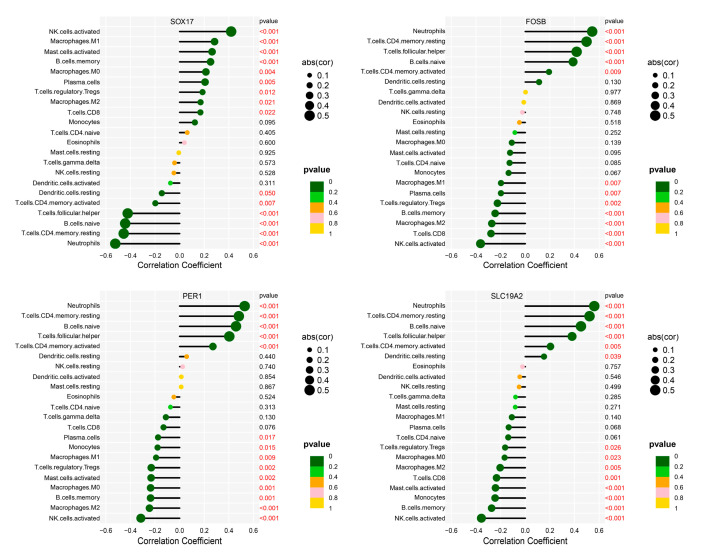
Correlation of hub genes with infiltrating immune cells in IgAN. In IgAN, the changes in immune cell abundance showed the same trend as the hub genes.

The analysis of immune cell infiltration in the glomerulus of IgAN, coupled with the identification of hub genes, offers valuable insights into the pathogenesis of the disease. The findings indicated that specific immune cell types are associated with the expression of hub genes, which exhibited altered expression patterns in IgAN (Figure 6). Notably, immune cells which were significantly reduced in the IgAN group, such as activated CD4+ memory T cells, follicular helper T cells, naive B cells, neutrophils, and resting CD4+ memory T cells, have the same tendency to change as the down-regulated hub genes FOSB, SLC19A2, and PER1. This correlation suggests that the decreased presence of these immune cells may be linked to the downregulation of these hub genes in IgAN. Conversely, immune cells that were significantly elevated in IgAN, including memory B cells, M0 macrophages, regulatory T cells (Tregs), CD8+ T cells, plasma cells, activated mast cells, activated NK cells, M1 macrophages, and monocytes, have the same tendency to change as the up-regulated hub gene SOX17. This positive correlation implies that the increased infiltration of these immune cells in IgAN may be connected to the upregulation of SOX17. The parallel trend of immune cells and hub genes underscores the strong association between immune cell infiltration and hub genes expression in IgAN.

These findings not only offer valuable insights into the pathogenesis of IgAN but also suggest potential targets for therapeutic intervention. By modulating the expression of these hub genes or targeting specific immune cells, it may be possible to regulate the immune response and slow the progression of IgAN. Given the limitations of this algorithm, the correlation between hub genes and immune cells may not be causal and future studies should validate these findings and investigate potential therapeutic strategies based on these observations.

## 4. Discussion

IgAN is a prevalent kidney disease characterized by a chronic and often variable course. The condition is marked by the deposition of IgA-containing immune complexes in the glomeruli, leading to inflammation and subsequent kidney damage. Diagnosis of IgAN typically relies on renal biopsy, which helps assess disease activity and prognosis. The pathogenesis of IgAN is multifaceted and not fully understood. The complex interactions of genetic, environmental, and immunological factors likely play an essential role in its development. The identification of hub genes and their association with specific immune cell types provide new insights into the underlying mechanisms of IgAN. However, more research is needed to fully understand the precise role of these genes and immune cells during disease progression.

Our study compared the transcriptome profiles of IgAN patients with healthy controls to identify differentially expressed genes (DEGs). KEGG analysis identified several key pathways, including the IL-17 signaling pathway, Th1 and Th2 cell differentiation, and Complement and coagulation cascades which are associated with IgAN. The IL-17 signaling pathway is closely linked to IgAN [26]. Upon IL-17 activation, FOSB dimerizes with the JUN protein to form the AP—1 complex regulating various physiological and pathological processes. Within this pathway, FOSB plays a pivotal role in modulating inflammatory and immune responses by controlling the transcription of downstream target genes. Inhibition of AP-1 has been shown to reduce the release of inflammatory mediators [27]. In addition, studies have demonstrated an imbalance in helper T cell subsets (Th1/Th2) in patients with IgAN, characterized by a decreased proportion of Th1 cells and an increased proportion of Th2 cells [28], thereby disrupting the Th1/Th2 ratio. This dysregulation may contribute to the onset and progression of renal interstitial fibrosis [29].

To identify hub genes associated with IgAN, we first applied weighted gene co-expression network analysis (WGCNA) to narrow down significant gene modules, followed by LASSO and RF machine learning algorithms to screen candidate genes, which can help us accelerate risk stratification and treatment matching in multiple diseases [30,31]. A consensus analysis of the two machine learning models revealed the four hub genes: FOSB, PER1, SLC19A2, and SOX17. Notably, FOSB has been consistently supported by previous studies [21,22,23,32,33,34,35,36,37], thereby validating the robustness of our screening strategy, which integrates multiple GEO datasets to enhance biomarker discovery. ROC curve analysis further confirmed the strong diagnostic potential of these hub genes in both training and testing sets. When combined, the four genes achieved an area under the curve (AUC) of 0.999 in the training set and 0.969 in the testing set, underscoring the accuracy of our selection and their promise as potential diagnostic biomarkers and therapeutic targets.

FOSB is a key component of the AP-1 complex [38] and is strongly associated with IgAN-related cellular functions, progressive renal injury and renal fibrosis [39,40,41]. It plays a pivotal role in complement activation [42] and inflammation [43], consistent with our KEGG analysis, and may influence immune cell recruitment and activation in the glomerular microenvironment.

PER1 (period circadian protein homolog 1) is a core regulator of circadian rhythms. Its knockout in Dahl salt-sensitive rats results in reduced creatinine clearance and aggravated kidney injury [44]. Beyond circadian regulation, PER1 modulates immune cell activity, showing a negative correlation with pro-inflammatory M1 macrophages and regulatory T (Treg) cells. Circadian disruption suppresses PER1 expression in mice, promoting M1 macrophage polarization [45]. In IgAN, PER1 downregulation may impair the suppression of macrophage-driven inflammation, leading to excessive M1 activation, increased pro-inflammatory cytokine release, and diminished Treg-mediated immunoregulation [46].

SLC19A2, also known as thiamine transporter 1 (THT1), mediates the cellular uptake of thiamine. Thiamine supplementation can reduce urinary albumin excretion by reversing metabolic dysfunction in glomerular endothelial cells, podocytes, and tubular epithelial cells, and protects against oxidative stress-induced injury to mesangial cells and the glomerular basement membrane [47,48]. While its direct role in immune regulation in IgAN remains unclear, its antioxidant and metabolic effects may indirectly modulate immune cell activation by limiting ROS driven inflammatory signaling.

SOX17 encodes a transcription factor involved in kidney development, oligodendrocyte differentiation, tumor cell–macrophage interactions, and negative regulation of the WNT signaling pathway [49,50]. Immune infiltration analysis revealed increased CD8^+^ T cells and Treg cells in IgAN, with SOX17 expression in glomeruli positively correlating with these immune populations [51]. Excessive ROS production in glomeruli can drive mesangial cell proliferation and extracellular matrix deposition [52,53]. Abnormal IgA deposition induces ROS-related mechanical stress, upregulating SOX17 expression [54], which may promote recruitment of CD8^+^ T cells and other inflammatory cells, further amplifying immune-mediated kidney injury [55]. These four hub genes may affect the occurrence and development of IgAN in handling ROS and immune regulation disorders.

Immune infiltration analysis of GEO data further revealed altered immune landscapes in IgAN, with increased memory B cells, naïve CD4^+^ T cells, and CD8^+^ T cells, and decreased activated dendritic cells, activated CD4^+^ memory T cells, follicular helper T cells, naïve B cells, neutrophils, and resting CD4^+^ memory T cells. Correlation analysis showed that upregulated hub genes were positively associated with increased immune cell levels, whereas downregulated hub genes correlated with reduced immune cell populations. For example, reduced activated CD4^+^ memory T cells were positively associated with downregulated FOSB, PER1, and SLC19A2. Given the central role of CD4^+^ T cells in orchestrating B cell responses, these findings highlight the immunological significance of the identified hub genes in IgAN [56].

## 5. Conclusions

By integrating multiple IgAN GEO microarray datasets and removing batch effects, our study uncovered previously unrecognized hub genes and signaling pathways. The combined transcriptomic and immune infiltration analyses provide new insights into the immune-related mechanisms of IgAN and offer a valuable reference for future prevention and treatment strategies. These findings deepen our understanding of IgAN pathogenesis and establish a scientific basis for developing novel therapeutic approaches, demonstrating the advantages of our study in data integration, predictive modeling, and immune mechanism exploration. Indeed, our study also has certain limitations. Considering that the results are all from bioinformatics analysis, subsequent experimental verification can further enhance the reliability of the conclusions.

## Data Availability

The datasets used and/or analyzed during the current study are available from the corresponding author on reasonable request.

## Abbreviations

The following abbreviations are used in this manuscript:

IgAN	IgA nephropathy
DEGs	differentially expressed genes
GEO	Gene Expression Omnibus
GO	Gene Ontology
WGCNA	weighted gene co-expression network analysis
AUC	area under the receiver operating characteristic curve
GS	gene significance
MM	module membership
LASSO	Least Absolute Shrinkage and Selection Operator
RF	random forest
PCA	principal component analysis
BP	biological process
CC	cell composition
MF	molecular function
